# Molecular determinants for α-tubulin methylation by SETD2

**DOI:** 10.1016/j.jbc.2021.100898

**Published:** 2021-06-19

**Authors:** Sarah Kearns, Frank M. Mason, W. Kimryn Rathmell, In Young Park, Cheryl Walker, Kristen J. Verhey, Michael A. Cianfrocco

**Affiliations:** 1 Program of Chemical Biology, University of Michigan, Ann Arbor, Michigan, USA; 2 Life Sciences Institute, University of Michigan, Ann Arbor, Michigan, USA; 3 Department of Medicine, Division of Hematology and Oncology, Vanderbilt University Medical Center, Nashville, Tennessee, USA; 4 Department of Genetics, Vanderbilt University, Nashville, Tennessee, USA; 5 Center for Precision Environmental Health, Baylor College of Medicine, Houston, Texas, USA; 6 Department of Cell and Developmental Biology, University of Michigan, Ann Arbor, Michigan, USA; 7 Department of Biological Chemistry, University of Michigan, Ann Arbor, Michigan, USA

**Keywords:** tubulin code, SETD2, lysine methyltransferase, post-translational modification, protein methylation, tubulin, microtubule, ccRCC, clear cell renal cell carcinoma, CTD, C-terminal domain, CTT, C-terminal tail, H3K36me3, histone-3 lysine 36 trimethylation, K40, lysine 40, NaPi, sodium phosphate, PTMs, post-translational modifications, R1625C, pathogenic arginine-to-cysteine mutation at position 1625, R2510H, arginine-to-histidine mutation at position 2510, RNA Pol II, RNA polymerase II, SETD2, SET domain containing 2, SRI, Set2–Rpb1–interacting, TEV, tobacco etch virus, tSETD2, truncated form of WT SETD2, tSETD2-FLAG, FLAG-tagged version of tSETD2, αTAT, α-tubulin acetyltransferase, αTubK40, α-tubulin at K40, αTubK40me3, methylation at lysine 40 of α-tubulin, αβ-tubulin(αK40A), αβ-tubulin dimers with a mutation of K40 to alanine, αβ-tubulin(ΔαCTT), αβ-tubulin proteins containing truncations of the CTTs of α-tubulin, αβ-tubulin(ΔβCTT), αβ-tubulin proteins containing truncations of the CTTs of β-tubulin

## Abstract

Post-translational modifications to tubulin are important for many microtubule-based functions inside cells. It was recently shown that methylation of tubulin by the histone methyltransferase SETD2 occurs on mitotic spindle microtubules during cell division, with its absence resulting in mitotic defects. However, the catalytic mechanism of methyl addition to tubulin is unclear. We used a truncated version of human wild type SETD2 (tSETD2) containing the catalytic SET and C-terminal Set2–Rpb1–interacting (SRI) domains to investigate the biochemical mechanism of tubulin methylation. We found that recombinant tSETD2 had a higher activity toward tubulin dimers than polymerized microtubules. Using recombinant single-isotype tubulin, we demonstrated that methylation was restricted to lysine 40 of α-tubulin. We then introduced pathogenic mutations into tSETD2 to probe the recognition of histone and tubulin substrates. A mutation in the catalytic domain (R1625C) allowed tSETD2 to bind to tubulin but not methylate it, whereas a mutation in the SRI domain (R2510H) caused loss of both tubulin binding and methylation. Further investigation of the role of the SRI domain in substrate binding found that mutations within this region had differential effects on the ability of tSETD2 to bind to tubulin *versus* the binding partner RNA polymerase II for methylating histones *in vivo*, suggesting distinct mechanisms for tubulin and histone methylation by SETD2. Finally, we found that substrate recognition also requires the negatively charged C-terminal tail of α-tubulin. Together, this study provides a framework for understanding how SETD2 serves as a dual methyltransferase for both histone and tubulin methylation.

Microtubules are dynamic cytoskeletal polymers that maintain cell shape, serve as tracks for intracellular trafficking, provide a structural framework for cell division, and form the structural elements of cilia. How microtubules achieve their many varied cellular functions comes, in part, from a tubulin code of multiple isoforms of α- and β-tubulin dimers and varied post-translational modifications (PTMs) (1, 2, 3). For example, differentiated cells express varying amounts of α- and β-tubulin isotypes to perform specialized roles (4). In addition, within each cell, there are subpopulations of microtubules with PTMs that further regulate microtubule-based functions. Analogous to how the histone code directs chromatin function, the combination of tubulin isotypes and PTMs comprises a tubulin code that specializes microtubule function in cells.

One key chromatin modifier that contributes to both the histone and tubulin codes is SET domain containing 2 (SETD2). SETD2 is an SAM-dependent lysine methyltransferase, which on chromatin is responsible for histone-3 lysine 36 trimethylation (H3K36me3), a mark associated with gene transcription (5, 6). Loss of SETD2 is embryonic lethal in part because its ability to trimethylate H3K36 is nonredundant (7). Many cancers including kidney, lung, bladder, glioma, and leukemia have inactivating mutations in SETD2 (8, 9, 10, 11, 12). In clear cell renal cell carcinoma (ccRCC), *SETD2* is the second most frequently mutated gene, contributing to 10 to 15% of all ccRCC cases (6, 13, 14, 15, 16). For example, pathogenic arginine-to-cysteine mutation at position 1625 (R1625C), found within the catalytic SET domain, ablates methyltransferase activity and is associated with poor prognosis. Another mutation, arginine-to-histidine at position 2510 (R2510H), occurs in the Set2–Rpb1–interacting (SRI) domain at the C terminus of SETD2 but does not result in loss of H3K36me3 (17, 18, 19), suggesting that pathogenicity associated with this SRI domain mutation is not due to loss of histone methylation.

Our previous work demonstrated that SETD2 can methylate tubulin and that methylation occurs at lysine 40 of α-tubulin (αTubK40me3) (19). In dividing cells, αTubK40me3 localizes to the minus ends of microtubules that form the mitotic spindle (19). Loss of SETD2 in ccRCC and KO of SETD2 in cells resulted in genomic instability and mitotic defects such as multipolar spindles, lagging chromosomes during anaphase, chromosome bridging during cytokinesis, and micronuclei (19, 20). These phenotypes correlated with a drastic reduction in both H3K36me3 and αTubK40me3 methylation. Reintroduction of a truncated form of human WT SETD2 (tSETD2) containing the SET and SRI domains rescued both histone and tubulin methylation as well as the mitotic defects (18, 19). In contrast, expression of tSETD2 with the R2510H mutation in the SRI domain rescued histone methylation but was unable to rescue tubulin methylation or the mitotic defects (18, 19), suggesting that a loss of SETD2 activity can result in increase of mitotic defects in a tubulin-dependent manner.

Many aspects of SETD2 function are still unexplained, such as how SETD2 recognizes and differentiates between methylation substrates. Structural information has been difficult to obtain, given the large size of SETD2 and the presence of multiple unstructured regions; to date, only two domains have been structurally characterized, the SET domain by crystallography (PDB ID: 4H12) and the SRI domain by NMR (PDB ID: 2A7O) (21, 22). Here, we took a biochemical reconstitution approach to define the minimal components required to methylate tubulin *in vitro*. Using purified tSETD2 and recombinant human tubulin (23, 24, 25) enabled precise control of tubulin isotype and PTM state *in vitro* and allowed us to generate mutant versions of both proteins to probe site selectivity of SETD2 methylation. We demonstrate that tSETD2 is sufficient to methylate tubulin *in vitro* and has a higher activity toward tubulin dimers over microtubules. We find that αTubK40, the same site that can be acetylated (26, 27, 28, 29), is the only site methylated by tSETD2. We also find that the SRI domain of SETD2 is important for binding tubulin substrate as the tSETD2–R2510H mutant can neither pull down nor methylate tubulin. Because the SRI domain of SETD2 makes protein–protein interactions with RNA polymerase II (RNA Pol II) during transcription (22), we investigated residues that could distinguish binding between tubulin and RNA Pol II. We found that positively charged residues within the SRI domain are more important for tubulin binding where aromatic residues are more critical for RNA Pol II binding. In addition, we found that SETD2 recognizes the negatively charged C-terminal tail (CTT) of α-tubulin, likely through electrostatic interactions. Together, this work further establishes SETD2 as a tubulin methyltransferase and provides a molecular basis for changes in tubulin and histone methylation derived from ccRCC mutations in SETD2 because of SRI-domain regulation.

## Results

### SETD2 methylates tubulin *in vitro*

**Figure 1:**
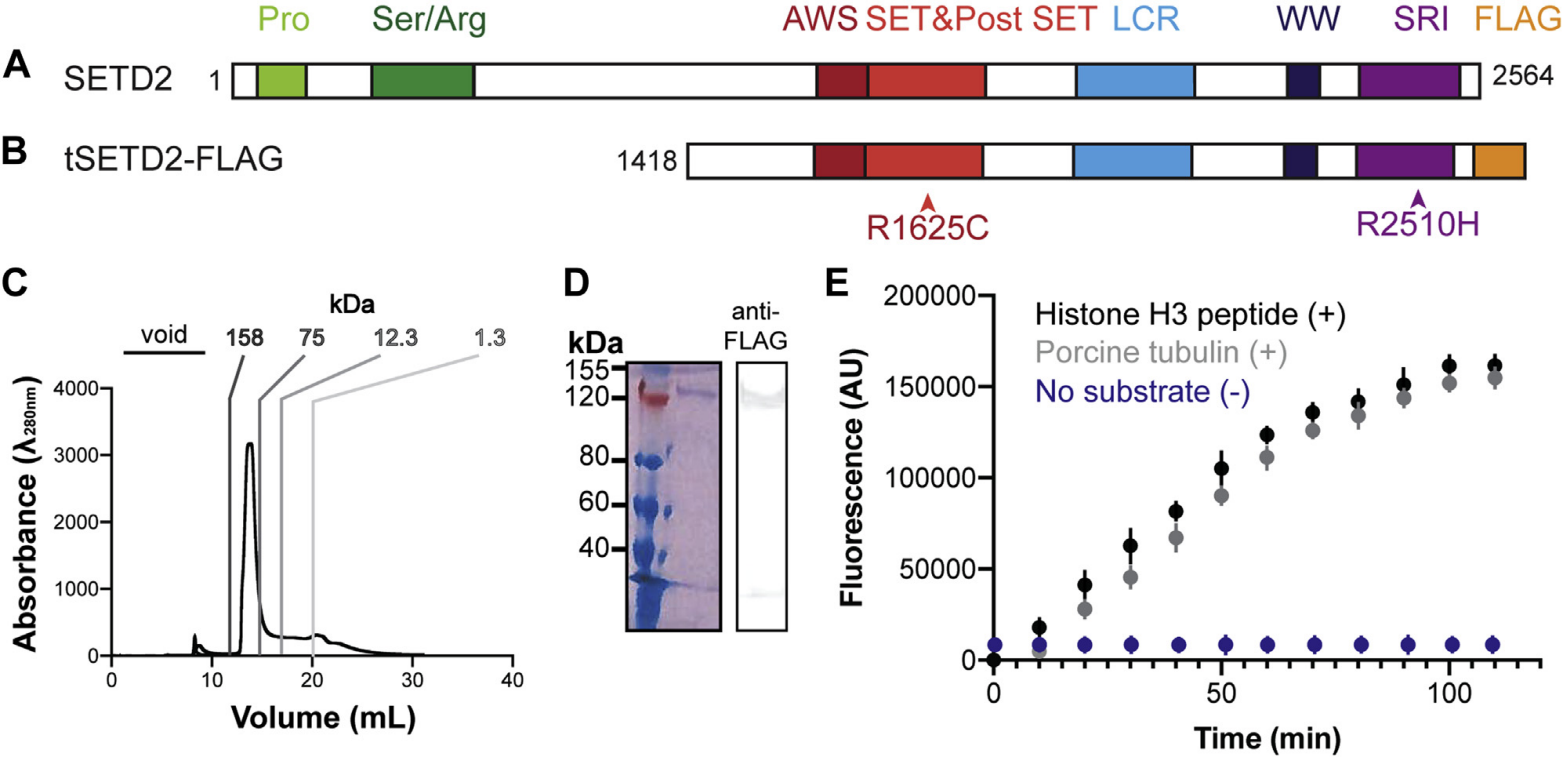
**tSETD2-FLAG methylates tubulin *in vitro*.** Schematic of (*A*) full-length and (*B*) tSETD2-FLAG domain organization. Pro (*light green*), Ser/Arg (*dark green*), AWS (*dark red*), SET (*red*), LCR (*light blue*), WW (*dark blue*), SRI (*purple*), FLAG-tag (*yellow*). *C* and *D*, tSETD2-FLAG was purified from HEK293 cells and analyzed by (*C*) size-exclusion chromatography and (*D*) Coomassie-stained gel (*left*) and anti-FLAG Western blot (*right*). *E*, fluorescence-based assay of methyltransferase activity over time. 2 μM tSETD2-FLAG was incubated with the methyl donor SAM and 5 μM of either histone H3 peptide (aa 21–44, *black*) or porcine brain tubulin (*gray*) substrate. Automethylation (negative control) of tSETD2-FLAG with SAM, but no substrate is shown in *blue*. These background values were subtracted from the histone peptide and porcine tubulin reactions before plotting the graph; hence, absorbance units (AU) = 0 at time 0 for both histone peptide and porcine tubulin traces. Data are presented as an average ± SD of n = 4 experiments with two different tSETD2-FLAG purifications. For some data points, the SD is smaller than the symbol. AWS, associated with SET; LCR, locus control region; Pro, proline-rich; Ser/Arg, serine/arginine-rich; SRI, Set2–Rpb1–interacting; tSETD2-FLAG, FLAG-tagged version of tSETD2; WW, tryptophan-rich.

To reconstitute tubulin methylation *in vitro*, we purified a truncated form of human WT SETD2 (tSETD2, aa1418–2564, Fig. 1, *A* and *B*) containing both SET and SRI domains. The smaller size of tSETD2 made it more amenable for biochemical purification, and previous work indicated that tSETD2 is sufficient to rescue SETD2 loss of function (18, 19). When expressed in mammalian cells, a FLAG-tagged version of tSETD2 (tSETD2-FLAG) localized to the nucleus during interphase and was dispersed throughout mitotic cells (Figs. S1 and S2), similar to the localization of expressed full-length and endogenous SETD2 (30, 31). Recombinant tSETD2-FLAG was purified from HEK293 cells, yielding a single species on size-exclusion chromatography (Fig. 1*C*) and a single band detected by SDS-PAGE (Fig. 1*D*). We confirmed that this band was SETD2-FLAG *via* Western blot against the FLAG epitope (Fig. 1*D*).

To measure the enzymatic activity of tSETD2-FLAG, we adopted a fluorescence-based assay that monitors S-adenosyl homocysteine production, the product of SAM-dependent methyl transfer. In this assay, an increase in fluorescence directly corresponds to tSETD2 activity (Fig. 1*E*). Thus, tSETD2-FLAG was incubated with porcine brain tubulin protein and the methyl donor SAM. As a positive control, we used an H3 peptide (residues 21–44) that can be methylated by tSETD2 (32). As a negative control, we measured fluorescence activity of tSETD2-FLAG in the presence of SAM but the absence of any substrate. In this case, any methyltransferase activity is indicative of automethylation that can then be subtracted out of substrate-containing reactions. Both histone peptide and tubulin protein substrates produced an increase in fluorescence activity over time (Fig. 1*E*), indicating that the reconstituted tSETD2-FLAG protein has activity toward tubulin substrate.

### tSETD2 displays a higher activity toward tubulin dimers than microtubule polymer

**Figure 2:**
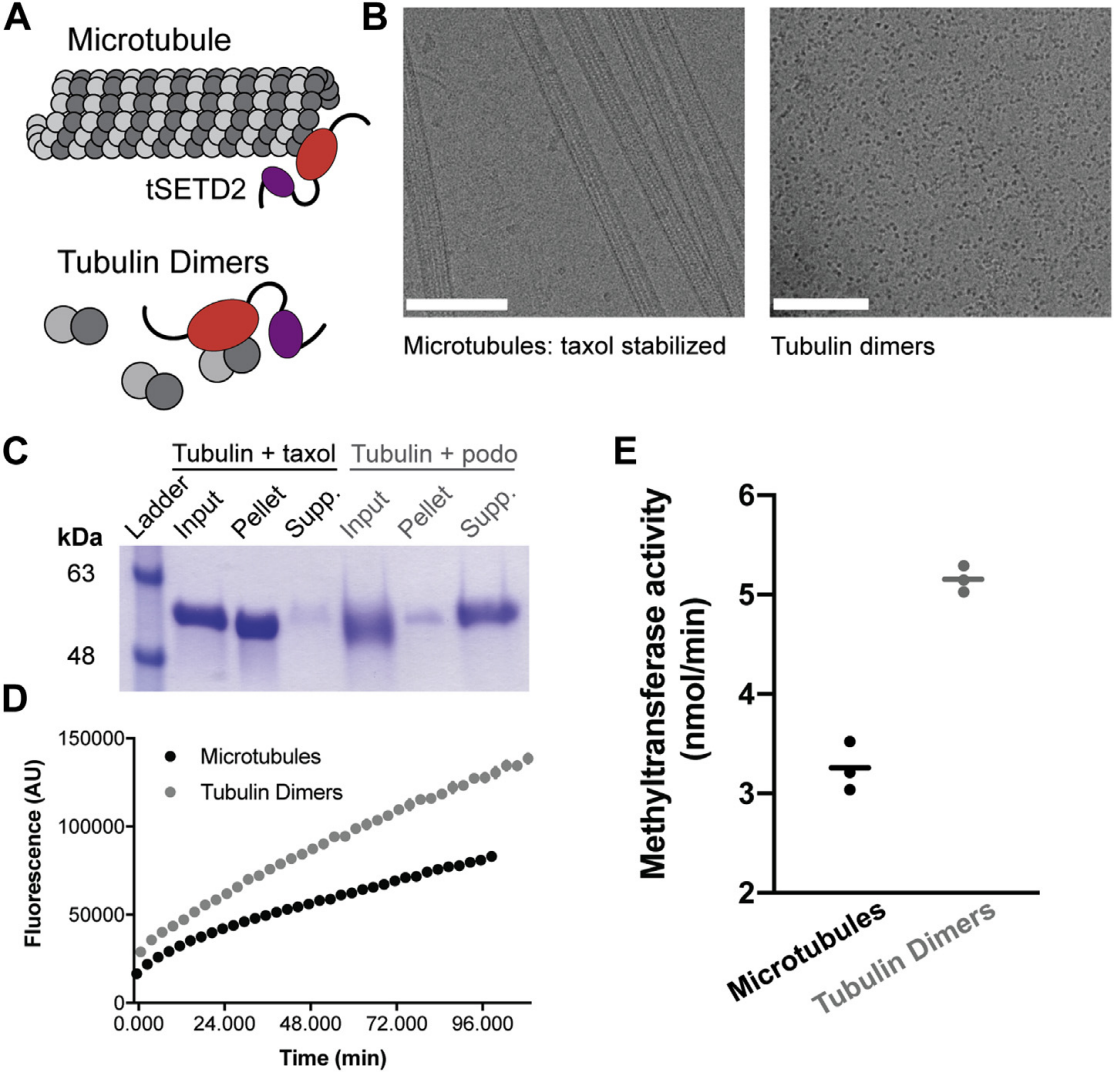
**tSETD2-FLAG has higher activity toward tubulin dimers than microtubule polymer.***A*, schematic showing tSETD2 (enzymatic SET domain in *red* and SRI domain in *purple*) methylation on either tubulin dimers or polymerized microtubules. *B* and *C*, verification of the polymer state. Porcine tubulin was polymerized and stabilized with taxol or treated with podophyllotoxin (podo) to prevent polymerization. *B*, micrographs of (*left*) taxol-stabilized microtubules and (right) tubulin dimers in a Tris-based buffer to prevent polymerization (see Experimental procedures). The scale bar represents 100 nm. *C*, microtubules were pelleted by centrifugation, and the pellet and supernatant fractions were separated by SDS-PAGE and stained with Coomassie. *D* and *E*, methyltransferase assay. 2 μM of purified tSETD2-FLAG was incubated with SAM and 5 μM porcine brain tubulin dimers (with podophyllotoxin, *gray*) or microtubules (with taxol, *black*) substrates, and methyltransferase activity was monitored over time. *D*, representative fluorescence traces of tSETD2-Flag activity. *E*, each *dot* indicates the methyltransferase activity measured in a single experiment, and error bars show the average ± SD across a total of three experiments. SRI, Set2–Rpb1–interacting; tSETD2-FLAG, FLAG-tagged version of tSETD2.

Our previous work showed that glutathione-S-transferase-tagged SETD2 (1392–2564) was capable of methylating tubulin in both soluble dimeric and polymerized microtubule states (19); however, the relative ability of SETD2 to methylate these substrates was not tested. To do this, we measured tSETD2-FLAG activity against microtubules maintained in a polymerized state with taxol as compared with porcine brain tubulin dimers maintained in an unpolymerized state with podophyllotoxin (33, 34) (Fig. 2, *A*–*C*). We found that tSETD2-FLAG has higher activity toward tubulin dimers at 5 μM (5.08 ± 0.06 nmol/min) than polymerized microtubules at the same concentration (3.19 ± 0.05 nmol/min) (Fig. 2, *D* and *E*). This finding is consistent with the K40 methylation site on α-tubulin, which is luminal in polymerized microtubules, being more accessible in a tubulin dimer context. Microtubule methylation may then occur *via* the incorporation of methylated dimers into microtubules.

### SETD2 does not methylate sites other than α-tubulin at K40

We previously determined that SETD2 can methylate α-tubulin at K40 (αTubK40) using recombinant glutathione-S-transferase-tagged SETD2 (1392–2564) and porcine brain tubulin followed by MS (19). However, brain tubulin contains numerous isotypes and premodified tubulin proteins, and thus, it has been difficult to determine whether there are additional sites for SETD2 methylation on α- or β-tubulin.

**Figure 3:**
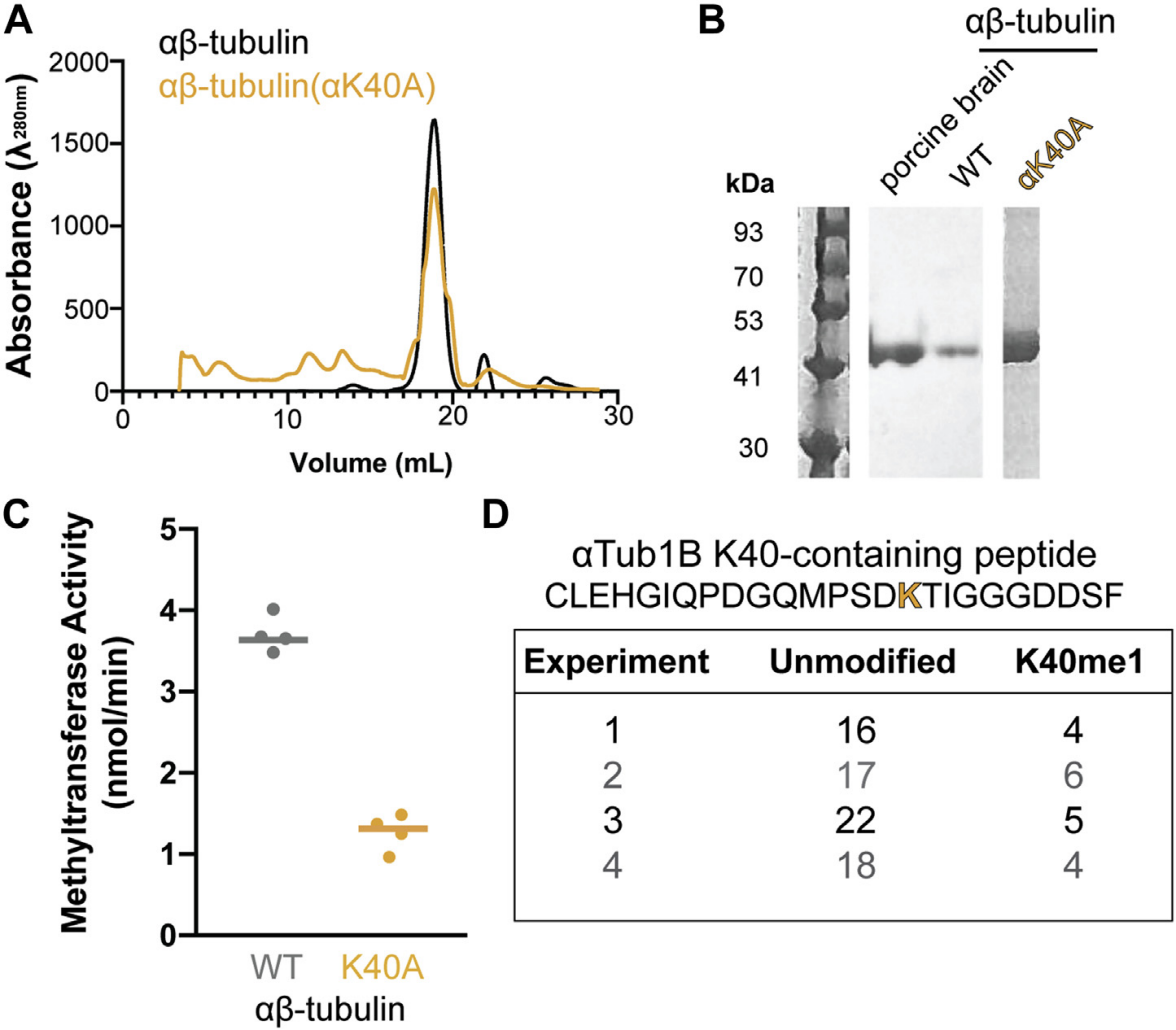
**αTubK40 is the primary site for methylation by tSETD2.***A* and *B*, recombinant single-isotype αTubA1B-βTub3B (αβ-tubulin) proteins, both WT (*black*) and the αK40A (*yellow*) mutant, were purified from Hi Five insect cells and analyzed by (*A*) size-exclusion chromatography and (*B*) Coomassie-stained SDS-PAGE gel. *C*, methyltransferase activity of 2 μM of tSETD2-FLAG incubated with 5 μM WT αβ-tubulin or mutant αβ-tubulin(αK40A) recombinant tubulins. Each *dot* indicates the methyltransferase activity from a single experiment, and the *bar* indicates the average value across n = 4 experiments. *D*, MS analysis of tubulin methylation. The table lists the sequence of the only peptide found to be methylated on lysine in the presence of both tSETD2-FLAG and SAM followed by the number of unmodified or K40 monomethylated peptides identified in each experiment. The raw MS data have been deposited at: https://osf.io/m62x7/. αTubK40, α-tubulin at K40; K40, lysine 40; tSETD2, truncated form of WT SETD2; tSETD2-FLAG, FLAG-tagged version of tSETD2.

To determine if there are other methylation sites on tubulin, we utilized recombinant human tubulin. Recent advances in the expression and purification of recombinant single-isotype tubulin (23, 24, 25) allowed us to purify human αTub1B/βTub3 tubulin dimers (hereafter referred to as αβ-tubulin) from insect cells using the baculovirus expression system (Fig. 3, *A* and *B*). This system allowed us to mutate the known methylation site K40 on α-tubulin by purifying αβ-tubulin dimers with a mutation of K40 to alanine [αβ-tubulin(αK40A)]. We confirmed that αβ-tubulin, both WT and K40A, were functional by observing their ability to polymerize into microtubules from GMPCPP-tubulin seeds (Fig. S3).

The ability of tSETD2-FLAG to methylate WT αβ-tubulin *versus* mutant αβ-tubulin(αK40A) was then measured using the methyltransferase assay. We found that the tubulin methyltransferase activity of tSETD2-FLAG was decreased when provided with the mutant αβ-tubulin(αK40 A) as a substrate (1.28 ± 0.19 nmol/min) as compared with the methyltransferase activity toward WT αβ-tubulin (3.73 ± 0.04 nmol/min) (Fig. 3*C*). This result suggests that although αTubK40 is the major site for methylation by tSETD2, there may be additional methylation sites on α- or β-tubulin. To identify potential methylation site(s), we carried out MS analysis of methyltransferase reactions containing tSETD2-FLAG and WT or K40A mutant tubulins. As a negative control, experiments lacking the methyl donor SAM were carried out in parallel. For WT tubulin, we could only identify monomethylation on αK40 (Fig. 3*D*) and only in the presence of both tSETD2-FLAG and SAM. In the case of αK40A mutant tubulin, we were unable to identify any methylation sites on α- or β-tubulin (Fig. 3*D*) even in the presence of both tSETD2-FLAG and SAM. These results suggest that αTubK40 is the only site methylated by tSETD2-FLAG.

### An R2510H SRI-domain mutation alters tSETD2's ability to bind to and methylate tubulin

**Figure 4:**
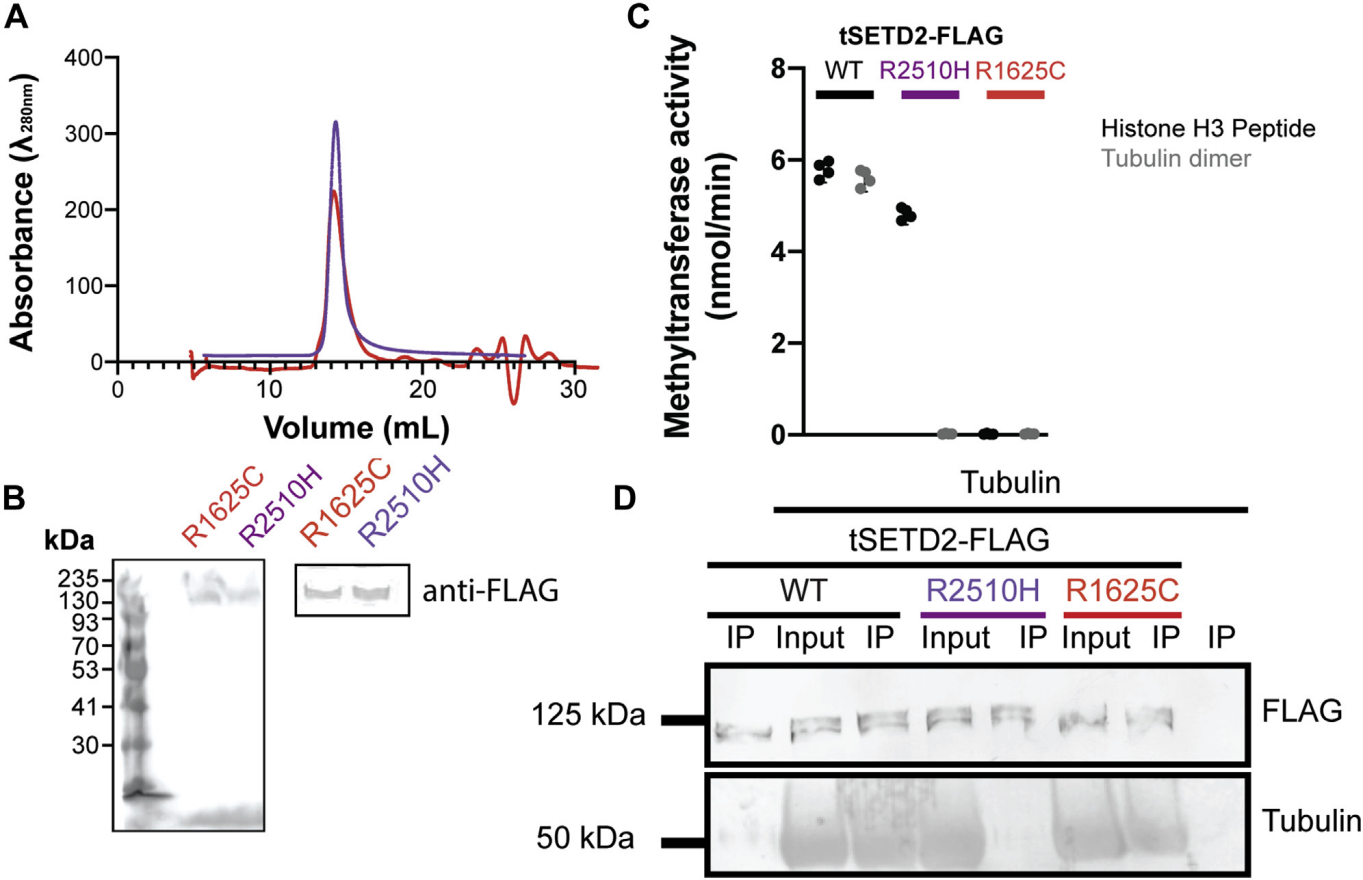
**ccRCC-associated R2510H mutation in the SRI domain blocks binding and methylation of tubulin.***A* and *B*, tSETD2-FLAG protein with mutation of R1625C (*red*) or R2510H (*purple*) was purified from HEK293 cells and analyzed by (*A*) size-exclusion chromatography and (*B*) Coomassie-stained gel (*left*) and anti-FLAG Western blot (*right*). *C*, methyltransferase activity of 2 μM WT, R1625C, or R2510H tSETD2-FLAG proteins against 5 μM histone-H3 peptide (*black*) or porcine brain tubulin (*gray*) substrates. Each *dot* indicates the methyltransferase activity measured in a single experiment across a total of four experiments. *D*, coimmunoprecipitation of tubulin protein with WT, R1625C, or R2510H tSETD2-FLAG proteins. The input and anti-FLAG pellet (IP) fractions were blotted with antibodies against the FLAG tag (*top*) and β-tubulin E7 (*bottom*). ccRCC, clear cell renal cell carcinoma; R1625C, pathogenic arginine-to-cysteine mutation at position 1625; R2510H, arginine-to-histidine mutation at position 2510; tSETD2-FLAG, FLAG-tagged version of tSETD2.

SETD2's methylation of tubulin is dependent on its SET and SRI domains (19, 20). A ccRCC mutation in the SET domain, R1625C, abolishes SETD2 catalytic activity, but the molecular basis for reduced methylation of tubulin by a ccRCC-associated mutation in the SRI domain (R2510H) remains unclear. To investigate this, we generated and purified tSETD2(R2510H)-FLAG from HEK293 cells (Fig. 4, *A* and *B*). As a control, we also generated and purified tSETD2-Flag containing a mutation in the catalytic SET domain, tSETD2(R1625C)-FLAG (Fig. 4, *A* and *B*). Using purified tSETD2 proteins and the methyltransferase assay, we confirmed that mutation of R1625C in the catalytic domain abolished the ability of tSETD2-FLAG to methylate both porcine brain tubulin protein and the control H3 peptide, whereas mutation of R2510H in the SRI domain only abolished the ability of tSETD2-FLAG to methylate tubulin protein (Fig. 4*C*).

We hypothesized that the SRI domain mutation reduced tubulin methylation by decreasing SETD2's ability to bind to tubulin. To test this, we performed coimmunoprecipitation assays to assess the interaction of WT, R1625C, and R2510H tSETD2-FLAG with brain tubulin. We found that both WT tSETD2-FLAG and the SET domain mutant tSETD2(R1625C)-FLAG bound to tubulin, but that the tSETD2(R2510H)-FLAG SRI-domain mutation abolished tubulin binding (Fig. 4*D*). These results suggest that (1) the ccRCC-associated mutation of R2510H in the SRI domain abolishes the ability of tSETD2 to bind to and methylate tubulin as a substrate and (2) the SRI domain is critical for interaction with tubulin as a substrate.

### Distinct residues in the SRI domain allow substrate selection for tubulin or RNA Pol II

**Figure 5:**
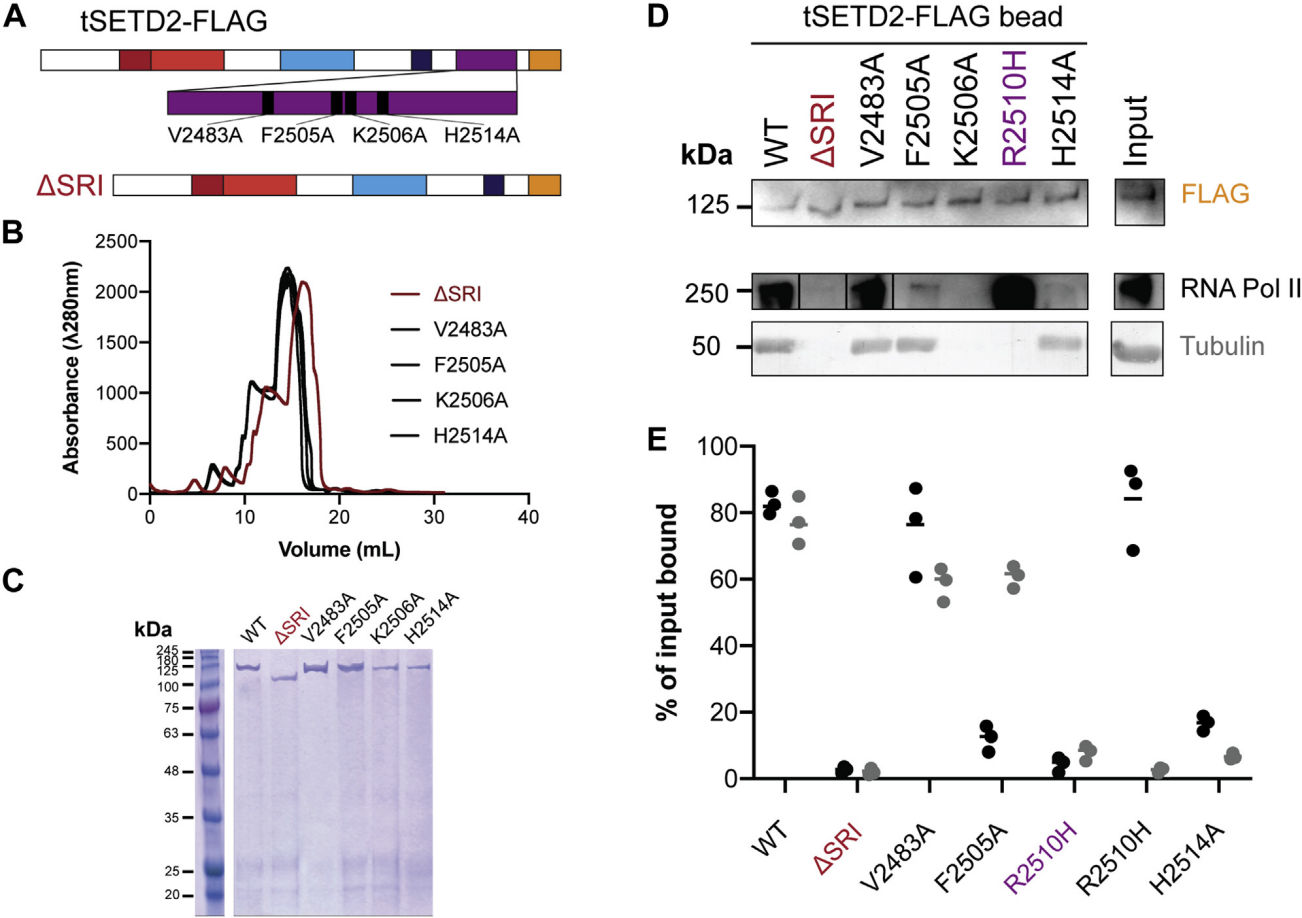
**Residues in the SRI domain of SETD2 distinguish tubulin and RNA-Pol II binding.***A*, schematic of point mutations or deletion of the SRI domain in tSETD2-FLAG. *B* and *C*, purification of tSETD2-FLAG containing mutations in or deletion of the SRI domain. The proteins were analyzed by (*B*) size-exclusion chromatography and (*C*) Coomassie-stained gel. *D* and *E*, purified WT, ΔSRI, or mutant versions of tSETD2-FLAG protein were bound to anti-Flag beads and then incubated with either tubulin protein or HEK293 cell lysate. *D*, the presence of tSETD2-FLAG variant, tubulin, and Pol II in the bead pellet was analyzed by Western blotting with antibodies to the FLAG tag, α-tubulin, and Pol II, respectively. The far-right column shows the input for the reaction with WT tSETD2-FLAG. *E*, quantification of RNA Pol II (*black*) and porcine brain tubulin (*gray*) copelleting with tSETD2-FLAG as a percentage of the input reaction. Each *dot* indicates the percent bound in a single experiment across a total of three experiments. RNA Pol II, RNA polymerase II; SRI, Set2–Rpb1–interacting; SETD2, SET domain containing 2; tSETD2-FLAG, FLAG-tagged version of tSETD2; ΔSRI, construct lacking the SRI domain.

The SRI domain of SETD2 has previously been shown to engage in protein–protein interactions with the highly phosphorylated C-terminal domain (CTD) repeat of RNA Pol II for recruitment of SETD2 to chromatin during transcription (22, 35). Thus, our finding that mutation of R2510H in the SRI domain abolishes tubulin binding (Fig. 4*D*) suggests that the SRI domain plays a major role in substrate recognition for both tubulin and RNA Pol II substrates. To test this, we took advantage of NMR-based studies that identified SRI residues impacted by binding to peptides mimicking the phosphorylated C terminus of RNA Pol II (22, 35). We targeted these residues and generated tSETD2-FLAG variants with alanine mutations to V2483, F2505, K2506, R2510, and H2514 (Fig. 5*A*). We also generated a construct lacking the SRI domain as a control (Fig. 5*A*). The mutant and deletion variants of tSETD2-FLAG were purified from mammalian cells (Fig. 5, *B* and *C*).

We tested the ability of the purified mutant and deletion tSETD2-FLAG proteins to bind to RNA Pol II and tubulin using the coimmunoprecipitation assay. The tSETD2-FLAG proteins bound to anti-FLAG beads were incubated with either porcine brain tubulin or HEK293 cell lysates containing endogenous RNA Pol II. As expected, deletion of the SRI domain abolished the ability of tSETD2-FLAG to bind to both RNA Pol II and tubulin substrates (Fig. 5, *D* and *E*). Interestingly, the SRI-domain mutants varied in their ability to bind to the two substrates. The V2483A mutant retained binding to both substrates, the F2505A retained tubulin but not RNA Pol II binding, the R2510H mutant retained RNA Pol II but not tubulin binding, and the K2506A and H2514A mutants lost the ability to bind to both tubulin and RNA Pol II (Fig. 5, *D* and *E*). These results indicate that the SRI domain is involved in substrate recognition and that distinct residues in the SRI domain contribute to recognition of different substrates.

### SETD2 recognizes tubulin *via* α-tubulin's CTT

**Figure 6:**
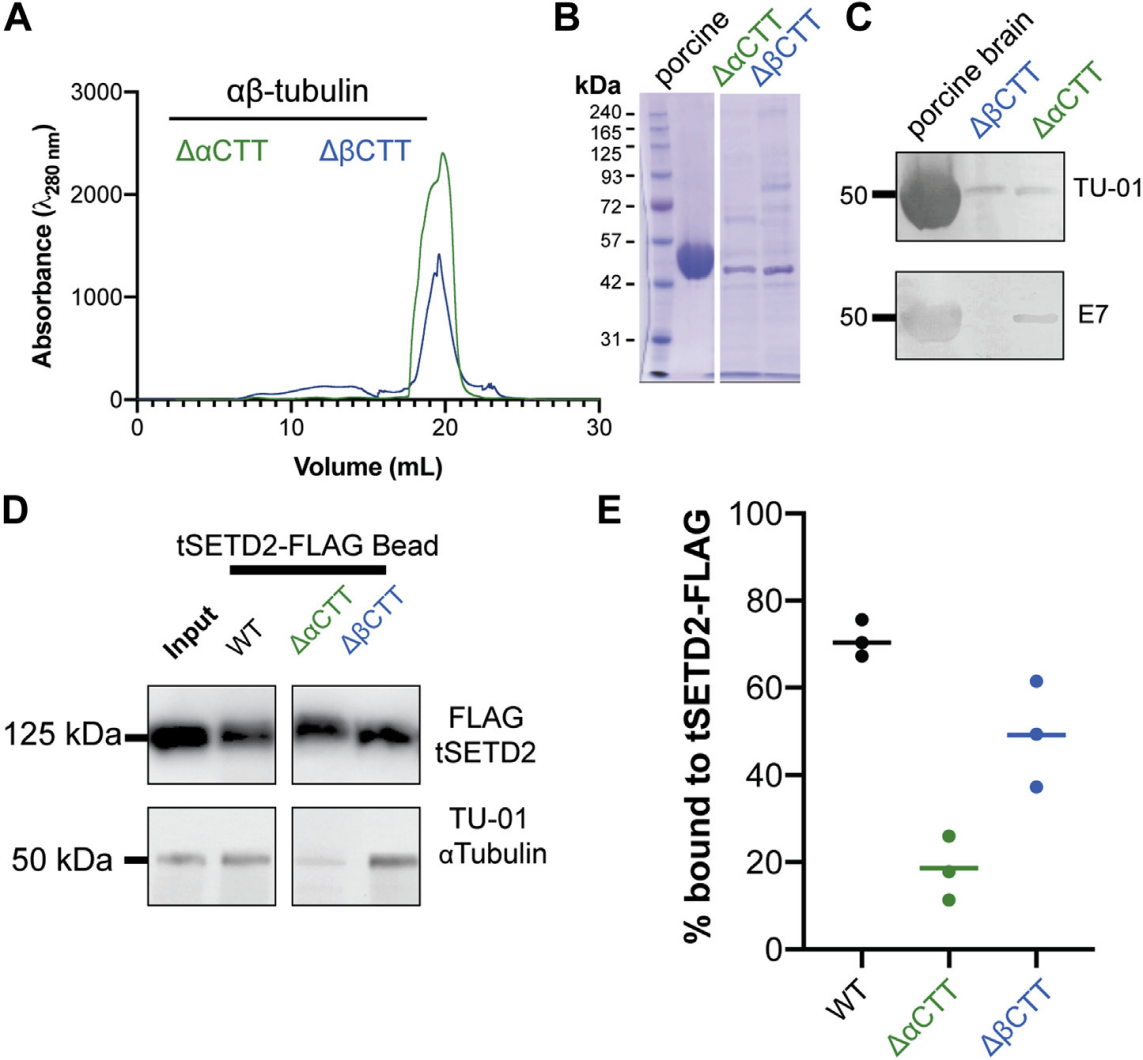
**tSETD2-FLAG binds to the C-terminal tail (CTT) of α-tubulin.***A* and *B*, tailless forms of recombinant tubulin, αβ-tubulin(ΔαCTT), and αβ-tubulin(ΔβCTT) were purified from Hi Five insect cells and analyzed by (*A*) size-exclusion chromatography and (*B*) Coomassie-stained gel. *C*, Western blot of porcine brain tubulin, purified αβ-tubulin(ΔαCTT), and purified αβ-tubulin(ΔβCTT) samples shown in *panel B* with antibodies that recognize the N terminus of α-tubulin (TU-01, *top*) or the CTT of β-tubulin (E7, *bottom*). *D* and *E*, coimmunoprecipitation of tSETD2-FLAG with WT recombinant αβ-tubulin, αβ-tubulin(ΔαCTT), or αβ-tubulin(ΔβCTT). *D*, representative Western blots of the immunoprecipitation pellets with antibodies to tSETD2-FLAG (FLAG, *top*) and the N terminus of α-tubulin (TU-01, *bottom*). The far-left column shows the input for the reaction with WT tubulin. *E*, quantification of the amount of tubulin copelleting with tSETD2-FLAG compared with the input. Each *dot* represents the percent bound in one experiment, and the *line* represents the average across a total of three experiments. tSETD2-FLAG, FLAG-tagged version of tSETD2.

The interaction between SETD2 and RNA Pol II involves the negatively charged phosphorylated C-terminal repeat domains of Pol II (22, 35). This led us to hypothesize that SETD2 recognition of tubulin involves similar charge–charge interactions, particularly between positively charged residues in the SETD2 SRI domain (Fig. 5, *D* and *E*) and the negatively charged CTTs of α- and/or β-tubulin. To test this, we used the recombinant single-isotype tubulin system to generate αβ-tubulin proteins containing truncations of the CTTs of either α-tubulin [αβ-tubulin(ΔαCTT)] or β-tubulin [αβ-tubulin(ΔβCTT)] and purified the tubulin dimers from insect cells (Fig. 6, *A* and *B*).

We tested the ability of tSETD2-FLAG to bind to the tailless tubulins using a coimmunoprecipitation assay. As most anti-tubulin antibodies recognize the CTTs, we first identified conditions for detecting the tailless tubulins by Western blotting. For this, we used a polyclonal antibody TU-01 whose epitope resides in the N terminus of α-tubulin. As expected, the TU-01 antibody was able to detect both αβ-tubulin(ΔαCTT) and αβ-tubulin(ΔβCTT) proteins by Western blotting, whereas an antibody against the CTT of β-tubulin (E7) failed to recognize the αβ-tubulin(ΔβCTT) protein (Fig. 6*C*). Immobilized tSETD2-FLAG was incubated with either αβ-tubulin(ΔαCTT) or αβ-tubulin(ΔβCTT) tubulin proteins. Whereas t-SETD2-FLAG pulled down porcine brain and αβ-tubulin(ΔβCTT) tubulins, it showed a reduced ability to coprecipitate αβ-tubulin (ΔαCTT) tubulin (Fig. 6, *D* and *E*). From these experiments, we conclude that tSETD2-FLAG binds to tubulin largely *via* the negatively charged CTT of α-tubulin.

## Discussion

**Figure 7:**
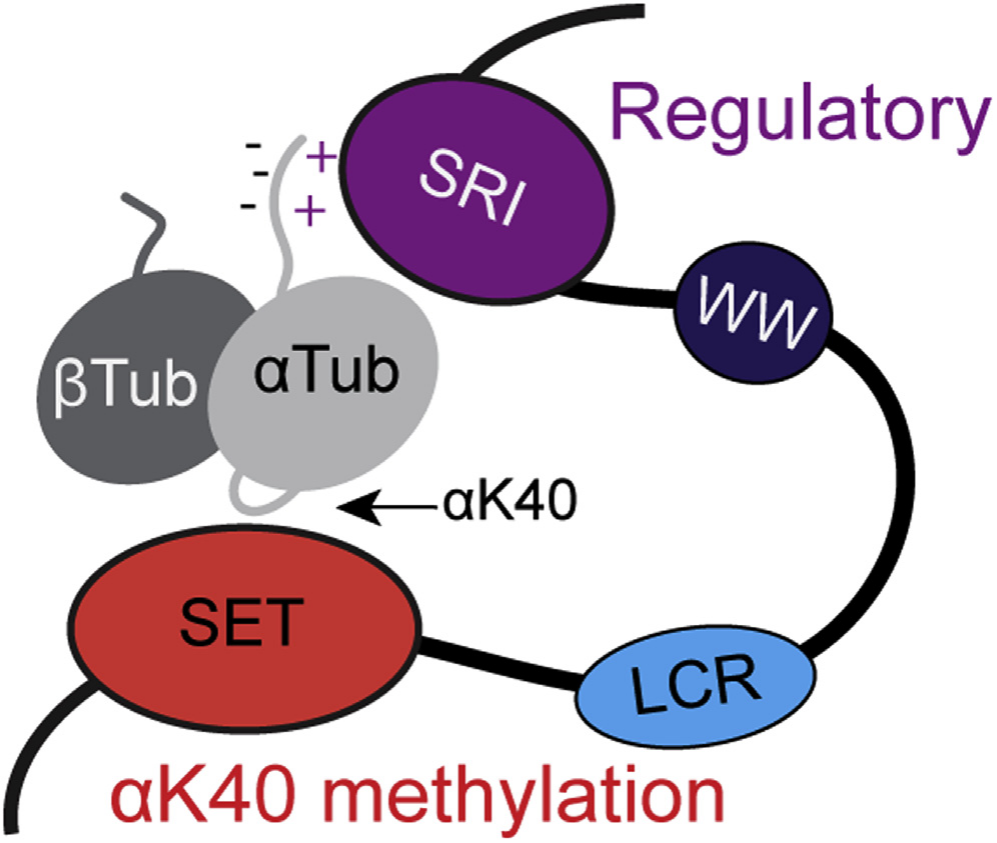
**Proposed model of tubulin methylation by tSETD2.** The SRI domain (*purple*) recognizes the negatively charged CTT of α-tubulin (*gray*) and positions the SET domain (*red*) for methylation of αtubulin at the K40. CTT, C-terminal tail; K40, lysine 40; tSETD2, truncated form of WT SETD2; SRI, Set2–Rpb1–interacting.

In this study, we utilized *in vitro* biochemical reconstitution with recombinant proteins to determine how SETD2 recognizes and methylates tubulin. By exploiting known tubulin-targeting agents, we found that SETD2 preferentially methylates the dimeric form of tubulin *versus* microtubule polymers. Interestingly, our work indicates that SETD2's SRI domain makes electrostatic interactions with the CTT of α-tubulin in a mechanism distinct from RNA Pol II targeting, suggesting that this interaction positions the SET domain for methylation of residue K40 of α-tubulin (Fig. 7). As structural information is presently only available for the SET domain (PDB ID: 4H12) and the SRI domain (PDB ID: 2A7O), a future direction will be to obtain structures of tSETD2 with its substrates.

### SETD2 methylation is restricted to K40 of α-tubulin

Our MS analysis identified α-tubulin K40 as the only detectable site of methylation by tSETD2 on the recombinant single-isotype αβ-tubulin. This confirms and extends the previous work where trimethylation was detected at this site in mammalian cells (19). In our MS experiments, we were only able to detect a monomethylation mark on αK40, whereas in cells, trimethylated tubulin was detected (19). The canonical SETD2 activity is with dimethylated histone substrates. Although this suggests that mono- or di-methylated tubulin could be a preferred substrate for SETD2, it is known that SETD2 can monomethylate substrates (36), but typically dimethylates or trimethylates substrates. As such, tubulin methylation may require priming by other methyltransferases before SETD2 makes its mark in cells.

Although mutation of K40A did not completely abolish methyltransferase activity measured in the fluorescence-based assay, we were unable to detect any other methylated peptides for α- or β-tubulin by MS. It seems unlikely that the residual methyltransferase activity comes from tSETD2 itself as the automethylation activity is subtracted from our measurements. It is possible that SETD2 is able to methylate another tubulin residue that we have been unable to detect in our MS analysis, perhaps because the methylated peptide does not ionize well or the amount is below the limit of detection. It is also possible that tSETD2-FLAG or a copurifying methyltransferase enzyme from HEK293 cells is able to methylate a contaminating protein that copurifies with tubulin from the insect cells and/or with tSETD2-FLAG from the HEK293cells. Further work will be required to discern between these possibilities.

### Crosstalk between acetylation and methylation of α-tubulin K40

The K40 residue resides on a flexible loop of α-tubulin that is located within the lumen of a polymerized microtubule (37, 38) and could be accessible to modifying enzymes when tubulin is in either the soluble dimer or microtubule form. However, given the large size of the SETD2 protein (∼290 kDa) and the restricted size of the microtubule lumen (∼17 nm diameter), it has been puzzling how SETD2 could access the K40 residue within the microtubule lumen (19). We demonstrate that tSETD2 has higher methylation activity toward soluble tubulin over microtubules (Fig. 2). Binding of SETD2 to soluble tubulin requires the SRI domain of tubulin and the CTT of α-tubulin, an interaction that likely positions the SET domain for methylation of K40.

The K40 residue of α-tubulin is also known to be acetylated by α-tubulin acetyltransferase (αTAT) (39, 40). Whether SETD2 and αTAT compete for access to the K40 residue has been unclear. Given our results demonstrating a higher activity of SETD2 toward soluble tubulin and recent work demonstrating that αTAT1 preferentially acetylates polymerized microtubules and enters the microtubule lumen (40, 41, 42, 43, 44), the two enzymes appear to work on tubulin in different contexts. Specifically, SETD2 preferentially methylates soluble tubulin, whereas αTAT preferentially acetylates polymerized tubulin. The interplay between these enzymes is likely to play a role in specific cellular events. For example, elevated levels of α-tubulin acetylation correlate positively with metastatic potential (45) and αTAT inhibits cancer cell motility (46), whereas SETD2-mediated tubulin methylation corresponds with genomic stability and correct mitotic spindle formation (19, 20).

Interestingly, both SETD2 (this work) and αTAT (40) display slow enzymatic rates toward the tubulin substrate *in vitro.* SETD2 also shows similar low k_cat_ values toward purified nucleosome substrate (32). In a histone methylating context, SETD2 is typically recruited by other protein complexes (e.g., IWS1, SPT6, and RNA Pol II) (47, 48), suggesting that substrate binding and enzyme kinetics could be higher in cells.

### The role of the SETD2 SRI domain in substrate binding

Previous work demonstrated that the ccRCC-associated mutation R2510H in the SRI domain abolished the ability of SETD2 to methylate tubulin but not histone H3 (18, 19). We provide a mechanism for this finding by demonstrating that the R2510H mutation abolishes the ability of tSETD2 to bind to tubulin (Figs. 4 and 5) but has no effect on its ability to bind to interact with RNA Pol II from cell lysate (Fig. 5). That the SRI domain is required for binding to tubulin appears to contradict previous work, suggesting that the SET domain is sufficient for tubulin binding (19). These discrepancies could be due to the use of different constructs and expression systems, as well as differences in experimental protocols. The fact that the interaction of RNA Pol II is less sensitive to the R2510H mutation than tubulin may be related to the sequence and charge of these binding segments. Specifically, the CTD of Pol II contains 22 heptapeptide (YSPTSS) repeats and is highly phosphorylated, whereas the CTT of α-tubulin contains a short sequence of negatively charged residues. Thus, the interaction between the SRI domain of SETD2 and RNA Pol II may better tolerate the R2510H mutation than the interaction of the SRI domain with α-tubulin. If so, caution should be taken when studying interactions with RNA Pol II CTD peptides containing fewer repeats, which could produce different results than using full-length RNA Pol II. Nevertheless, these results provide strong support for the hypothesis that genomic instability in ccRCC can be driven by tubulin-dependent functions of SETD2 contributed by the SRI domain.

By mutagenesis of the SRI domain based on structural information in the literature (22, 49), we were able to identify residues that further distinguish the ability of SETD2 to bind to tubulin *versus* Pol II. We demonstrate that the ability of tSETD2 to bind to tubulin requires not only the SRI domain residue R2510 but also K2506 (Fig. 5). In contrast, the ability of tSETD2 to bind Pol II is influenced by F2505, K2506, and H2514. These results are largely consistent with previous studies using NMR structures of the SRI domain and titration experiments with phosphorylated Pol II peptides that implicated residues V2483, F2505, K2506, R2510, and H2514 in forming part of the SRI-Pol II binding interface (22, 35, 50). However, although the isolated SRI domain with the R2510H point mutant showed decreased binding to RNA Pol II CTD peptides (22), our results suggest that R2510H does not decrease the ability of tSETD2-FLAG to pull down Pol II from cell lysates (Fig. 5*C*). These differences are likely due to differences in experimental conditions including the use of an isolated SRI domain *versus* tSETD2 and the use of Pol II peptides *versus* full-length CTD as mentioned above.

### Recognition of the α-tubulin CTT by SETD2

Our results also demonstrate that tSETD2 requires the CTT of α-tubulin, a negatively charged region of the protein, for binding (Fig. 6). This is interesting, given previous work demonstrating that the SRI domain of SETD2 interacts with the phosphorylated, and thus negatively charged, C-terminal repeat domains of Pol II (35, 49, 51). It is thus tempting to speculate that charge–charge interactions are key for recognition of substrates by the SRI domain of SETD2. However, actin has recently been shown to be methylated by SETD2 (52), and unlike tubulin and RNA Pol II, actin does not have a long, flexible negatively charged tail or loop within its structure. However, in these studies, actin methylation by SETD2 required other binding partners, namely the Huntingtin protein, HTT, and the actin binding adaptor protein HIP1R. Future work will be required to delineate the mechanism by which SETD2 recognizes actin and other substrates.

Although our results suggest that SETD2 does not compete with αTAT for modification of the K40 residue, it likely competes with other tubulin-interacting proteins and/or modifying enzymes that target the CTT. The flexible CTTs of tubulin subunits extend from the surface of the microtubule and form a negatively charged surface that appears to serve as a recognition site for a large number of microtubule-associated proteins. For example, tubulin tyrosine ligase makes critical electrostatic interactions with α-tubulin CTT residues E445, E446, and E447 to align the CTT within its active site (53). Although the density of the CTT was unresolved by both X-ray crystallography and cryo-EM in the VASH–SVPB complex (54, 55), electrostatic interactions were observed with a compound, epoY, that mimics the tubulin CTT residues (56). Similarly, the tubulin CTTs are essential for microtubule recognition by tubulin tyrosine ligase-like 7, an enzyme that adds glutamate chains to the CTTs of both α- and β-tubulin (57, 58). As such, our work adds SETD2 to the growing list of tubulin-modifying enzymes that recognizes tubulin CTTs. Furthermore, the glutamate chains added by tubulin tyrosine ligase-like enzymes increase the negative charge of the CTT and could therefore alter the binding and activity of SETD2 toward tubulin. Thus, future work will examine the crosstalk between tubulin PTMs.

### Implications in cancer pathologies

Our findings provide further support for a role of SETD2 in writing both the histone and tubulin codes. As mutations in SETD2 continue to be identified in a growing list of tumor types (6, 20), it will be important to discern the relative roles of histone *versus* tubulin methylation in contributing to the underlying mechanisms of particular cancer phenotypes. A lack of tubulin methylation has been documented to result in multipolar spindles, genomic instability, and formation of micronuclei (18, 19, 20). A better understanding of histone and tubulin methylation by SETD2 could also drive anticancer drug development and thus could provide new therapeutic targets to help cancer patients. Interestingly, the histone H4 lysine 20 methylating enzyme SET8, along with transcription factor LSF, has been identified as a modifier of α-tubulin at K311 although the cellular implication of loss of K311 methylation remains to be determined (59). Other tubulin PTMs have been shown to impact mitotic progression and may also underlie cancer phenotypes. Recent work has shown that a disruption of α-tubulin detyrosination leads to reduced chromosome congression and increased errors of kinetochore–microtubule attachment (60, 61, 62, 63). Further investigations into tubulin PTMs and their modifying enzymes are required to understand the nuanced interaction between histone and tubulin modifications in cells and the implications for cancer progression.

## Experimental procedures

### Plasmids

An active truncated SETD2 construct (1418–2564) with a FLAG affinity tag (tSETD2-FLAG) in the pInducer vector for mammalian expression was generated by the Walker Lab. Single isoform αTub1B/βTub3 plasmid cDNA encoding *Homo sapiens* α-tubulin 1B (αTub1B, NP_006073.2) and β-tubulin 3 (βTub3, NM_178012.4) in pFastBac Dual vector (Thermo Fisher 10712024) was obtained from the Kapoor laboratory for insect cell expression (25). Point mutations and domain deletions were generated using QuikChange site-directed mutagenesis with Q5 Polymerase (NEB). All plasmids were verified by DNA sequencing.

### Purification

#### SETD2

tSETD2-FLAG was transfected into HEK 293 FreeStyle cells with FectoPRO transfection reagent (Polyplus, 10118–444), and cells were harvested 48 h later at 5000 rpm for 15 min (Beckman JLA 8.1). The pellet was suspended in the lysis buffer (50 mM Hepes, pH 7.5, 50 mM MgCl_2_, 150 mM NaCl, cOmplete protease inhibitor tablet [Sigma Aldrich, 4693159001]), and cells were lysed with 20 strokes of a dounce homogenizer. The lysate was ultracentrifuged (Beckman Ti70 337922) at 40,000 rpm for 40 min, and the supernatant was filtered with 1.0-um glass fiber filter (Pall Laboratory) and incubated with FLAG M2 affinity beads (Sigma Aldrich) equilibrated in the lysis buffer for 3 h. Beads were rinsed with three column volumes of the wash buffer (50 mM sodium phosphate (NaPi), pH 7.2, 150 mM NaCl, 5 mM β-mercaptoethanol), three column volumes of the salt buffer (wash buffer with 500 mM NaCl), and again with the wash buffer before the elution buffer (wash buffer with 300 ng 3x-FLAG peptide [Sigma Aldrich]) was added and incubated with beads overnight. The eluent was then run over an ion-exchange column (DEAE Sepharose, GE Life Sciences) with a salt gradient of 0 to 75%, followed by size-exclusion chromatography (Superose 6 Increase 10/300, Fisher Scientific) with gel filtration buffer (50 mM NaPi, pH 7.2, 150 mM NaCl, 5 mM β-mercaptoethanol, 5% glycerol). Fractions were pooled and concentrated with an Amicon Ultra 100K MWCO centrifugal filter unit and snap-frozen in liquid nitrogen and stored at −80 °C.

#### αTub1B/βTub3 tubulin

The baculovirus expression plasmid encodes for αTub1B with an N-terminal 6x-His tag and tobacco etch virus (TEV) cleavage site and βTub3 with C-terminal TEV cleavage site and Strep tag. After purification *via* the His and Strep tags, the tags are removed *via* TEV cleavage, leaving an alanine-proline linker at the N terminus of αTub1B and a glycine-serine linker at the C terminus of βTub3 (25). Purification was as previously described (25, 64). Briefly, the Bac-to-Bac system (Life Technologies) was used to generate recombinant baculovirus in SF9 cells. High Five cells (Thermo Fisher, B85502), grown to three million cells/ml in Lonza Insect-XPRESS (Fisher Scientific, BW12-730Q), were infected with P3 viral stocks at 10 ml/L. Cells were cultured in suspension at 27 °C and harvested at 60 h after infection. The following steps were done on ice or at 4 °C. Cells were lysed in an equal volume of lysis buffer (50 mM Hepes, 20 mM imidazole, 100 mM KCl, 1 mM MgCl_2_, 0.5 mM β-mercaptoethanol, 0.1 mM GTP, 3 U/ml benzonase, 1× Roche Complete EDTA-free protease inhibitor, pH 7.2) by dounce homogenization (20 strokes), and the homogenate was centrifuged at 55,000 rpm in a Type 70 Ti rotor (Beckman Coulter) for 1 h. The supernatant was then filtered through a 0.22-μm membrane (Fisher Scientific, 09740113) and loaded onto a 5-ml HisTrap HP column (GE Life Science 17-5247-01) pre-equilibrated with the lysis buffer. The column was washed with 35-ml lysis buffer until the UV absorption reached baseline, and then, protein was eluted with the nickel elution buffer (1× BRB80 (80 mM Pipes, 1 mM MgCl_2_, 1 mM EGTA), 500 mM imidazole, 0.2 mM GTP, 2 mM β-mercaptoethanol, pH 7.2). The fractions containing proteins were pooled, diluted 3-fold with the lysis buffer, and loaded onto 5-ml StrepTrap HP column (GE Life Science 29-0486-53). The column was washed with 25-ml 66% lysis buffer +33% nickel elution buffer, 25 ml of wash buffer 1 (1× BRB80, 1 mM β-mercaptoethanol, 0.1 mM GTP, 0.1% Tween-20, 10% glycerol, pH 7.2), and 25 ml of wash buffer 2 (1× BRB80 1 mM β-mercaptoethanol, 0.1 mM GTP, 10 mM MgCl_2_, 5 mM ATP, pH 7.2). The bound protein was then eluted with 5-ml StrepTrap elution buffer (1X BRB80, 20 mM imidazole, 2 mM β-mercaptoethanol, 0.2 mM GTP, 3 mM desthiobiotin, pH 7.2). The StrepTrap eluate was mixed with 4 mg of previously purified TEV protease (∼8 mg/ml stored in 40 mM Hepes, 150 mM KCl, 30%(w/v) glycerol, 1 mM MgCl_2_, 3 mM β-mercaptoethanol, pH 7.5) and incubated for 2 h on ice. The TEV-digested protein solution was concentrated with an Amicon Ultra 50K MWCO centrifugal filter unit (Millipore UFC901024) to 2 ml and loaded onto a Superdex 200 Increase 10/300GL column equilibrated in the size-exclusion buffer (1X BRB80, 5%(w/v) glycerol, 0.2 mM GTP, 2 mM β-mercaptoethanol, pH 6.8). Tubulin was eluted at ∼15 ml and concentrated to >3 mg/ml with an Amicon Ultra 50K MWCO centrifugal filter unit. The purified tubulin was snap-frozen in liquid nitrogen and stored at −80 °C. Tail-less tubulin was eluted at ∼18 ml and concentrated with an Amicon Ultra 30K MWCO centrifugal filter unit but otherwise was purified the same way.

### Methyltransferase assay

The activity of tSETD2-FLAG constructs was measured using a Methyltransferase Fluorescence Assay Kit (Cayman Chemical, 700150). This enzyme-coupled assay continuously monitors SAM-dependent methyltransferase activity by generating a fluorescent compound, resorufin, from the reaction product AdoHcy. Reaction mixtures of SETD2 with the substrate were assayed in SETD2 gel filtration buffer (50 mM NaPi, pH 7.2, 150 mM NaCl, 5 mM β-mercaptoethanol, 5% glycerol). Fluorescence is analyzed with an excitation wavelength of 530 to 540 nm and an emission wavelength of 585 to 595 nm using a PHERAstar Plate Reader (BMG LABTECH). A standard curve of resorufin concentration and fluorescence was used to determine concentration-dependent fluorescence. The initial velocities of the reaction curves were obtained by linear regression and used to calculate methyltransferase activity (Prism Version 8.1.1). Methylation activity is plotted for single experiments over a total of n repeats.

### Dimer or microtubule stabilization

Microtubule polymerization was inhibited by the addition of 50 μM of podophyllotoxin provided by Dan Sackett (Millipore Sigma, P4405). Polymerized microtubules were made with 2.5 mg/ml porcine brain tubulin with 2 mM GTP and 2 mM MgCl_2_ in BRB80 buffer and incubated at 37 °C for 45 min 100 μl of 10 μM taxol in BRB80 was added for at least 30 min and incubated at 37 °C. Microtubules were then spun down at 15K rpm for 10 min at room temperature (RT) and then resuspended in 100 μM taxol in BRB80. Microtubule stocks were made at 2 mg/ml and then diluted to perform methyltransferase assay in BRB80.

### MS

Purified single isoform tubulin and tSETD2-FLAG were incubated at molar ratio of 5:1 with excess S-adenosylmethionine (Sigma Aldrich, 86867-01-8) for 2 h at RT. Cysteines were reduced by adding 50 μl of 10 mM DTT and incubating at 45 °C for 30 min. Samples were cooled to RT, and alkylation of cysteines was achieved by incubating with 65 mM 2-chloroacetamide, under darkness, for 30 min at RT. An overnight digestion with 1-μg sequencing grade, modified chymotrypsin (Sigma Aldrich, 11418467001) was carried out at 30 °C with constant shaking in a thermomixer. Digestion was stopped by acidification, and peptides were desalted using Sep-Pak C18 cartridges using the manufacturer's protocol (Waters). Samples were completely dried using Vacufuge. Resulting peptides were dissolved in 8 L of 0.1% formic acid/2% acetonitrile solution, and 2 L of the peptide solution was resolved on a nano-capillary reverse phase column (Acclaim PepMap C18, 2 micron, 50 cm, ThermoScientific) using a 0.1% formic acid/2% acetonitrile (buffer A) and 0.1% formic acid/95% acetonitrile (buffer B) gradient at 300 nl/min over a period of 180 min (2–22% buffer B in 110 min, 22–40% in 25 min, 40–90% in 5 min followed by holding at 90% buffer B for 5 min and re-equilibration with buffer A for 25 min). The eluent was directly introduced into Orbitrap Fusion Tribrid mass spectrometer (Thermo Scientific, San Jose, CA) using an Easy-Spray Source. MS1 scans were acquired at 120K resolution (automatic gain control target = 1 × 10^6^; max ion trap = 50 ms). Data-dependent collision–induced dissociation MS/MS spectra were acquired using Top speed method (3 s) after each MS1 scan (new chemical entity ∼32%; automatic gain control target 1 × 10^5^; max ion trap 45 ms). Proteins were identified by searching the MS/MS data against *Homo sapiens* (UniProt; 20,353 reviewed entries; downloaded on June 29, 2019) using Proteome Discoverer (v2.4, Thermo Scientific). Search parameters included MS1 mass tolerance of 10 ppm and fragment tolerance of 0.2 Da; two missed cleavages were allowed; carbamidomethylation of cysteine was considered fixed modification and oxidation of methionine and deamidation of asparagine and glutamine were considered as potential modifications. The false discovery rate was determined using Percolator, and proteins/peptides with a false discovery rate of ≤1% were retained for further analysis.

### Pull-down assay

FLAG M2 beads were blocked with 3% bovine serum albumin in PBS for 1 h and equilibrated in the reaction buffer (tSETD2-FLAG gel filtration buffer: 50 mM NaPi, pH 7.2, 150 mM NaCl, 5 mM β-mercaptoethanol, 5% glycerol). tSETD2-FLAG protein (WT or variants) was added at 20 μM with a putative binding partner for 2 h in the presence of SAM. For tubulin, 0.25 mg/ml of porcine tubulin (Cytoskeleton, Inc.) was used, and for RNA Pol II, 10 μl of HEK293 FreeStyle clarified lysate was used. Beads were spun down, and the supernatant was collected as the fraction of unbound substrate. The beads were then resuspended in the reaction buffer to the total reaction volume, and the same amount of supernatant and beads was added to SDS-PAGE gel. Analysis of binding was conducted by Western blot with the following antibodies: anti-FLAG (1:1000, A9469; Sigma Aldrich), anti-tubulin E7 (1:1000, AB_528499; DSHB) and/or TU-01 (1:1000, 625902; BioLegend), and anti-RNA Pol II (1:1000, ab193468; Abcam), with secondary antibody anti-mouse (1:1000, ADI-SAB-100-J; Enzo Life Science) or anti-rabbit (1:1000, ADI-SAB-300-J; Enzo Life Science), respectively. Binding was quantified by measuring the background-subtracted intensity of each band with Fiji ImageJ (65) as a fraction of the input intensity. Each experiment was performed three times, independently.

### Immunohistochemistry

COS7 (ATCC, CRL-1651) cells transiently expressing tSETD2-FLAG constructs (Lipofectamine 2000 and Opti-MEM) were fixed with 4% formaldehyde in PBS, treated with 50 mM NH_4_Cl in PBS to quench unreacted formaldehyde and permeabilized with 0.2% Triton X-100 in PBS. Subsequently, cells were blocked in the blocking solution (0.2% fish skin gelatin in PBS). Primary antibodies against tubulin (1:2000, AB_528499; DSHB) and FLAG (1:2000, ab205606; Abcam) and secondary antibodies were applied in the blocking solution at RT for 1 h each, washing in between with the blocking solution. Nuclei were stained with 10.9 μM 4′,6-diamidino-2-phenylindole and cover glasses were mounted in ProLong Gold Antifade Mountant (Life Technologies). Images were collected on an inverted epifluorescence microscope (Nikon TE2000E) equipped with a 60×, 1.40 numerical aperture oil-immersion objective and a 1.5× tube lens on a Photometrics CoolSnap HQ camera driven by NIS-Elements (Nikon) software.

### Microtubule polymerization

#### Recombinant tubulin

We first prepared α1B/β3 microtubule seeds. Tubulin was thawed, mixed with GMPCPP (final 1.5 mM), diluted to ∼1.5 mg/ml with 1XBRB80 + 5% glycerol. Aggregates were cleared by centrifugation at 90,000 rpm for 10 min at 4 °C (TLA-120.1; Beckman Coulter), and then, microtubules were polymerized by incubation of the supernatant at 37 °C for 30 min. The microtubules were pelleted at 90,000 rpm for 10 min at 37 °C (TLA120.1; Beckman Coulter) and re-suspended in warm (37 °C) 1XBRB80 supplemented with 1 mM tris (2-carboxyethyl)phosphine. Next, we used these microtubule seeds to polymerize GTP-bound α1B/β3-tubulin. Another aliquot of recombinant tubulin was thawed, diluted to a final concentration of ∼3 mg/ml (1XBRB80, 33% glycerol, 1 mM GTP), and cleared by centrifugation at 90,000 rpm for 10 min at 4 °C (TLA120.1; Beckman Coulter). The supernatant was incubated at 37 °C for 2 min and then mixed with GMPCPP seeds from the prior step and incubated at 37 °C for 30 min. The microtubules were pelleted by centrifugation at 90,000 rpm for 10 min at 37 °C (TLA120.1; Beckman Coulter). Microtubule pellets were rinsed twice with 100-μl warm (37 °C) EM buffer (1× BRB80, 1 mM DTT, 0.1 mM ATP, 0.05% Nonidet P-40) before suspending in 30-μl cold EM buffer and then incubated on ice for 1 h. After centrifugation at 90,000 rpm for 10 min at 4 °C, the supernatant containing depolymerized GDP-tubulin (∼2 mg/ml, measured by the Bradford assay) was mixed with GMPCPP (final 2 mM) and then incubated on ice for 10 min. After an incubation at 37 °C for 2 min, the protein solution was mixed with 30-μl warm (37 °C) EM buffer followed by 37 °C incubation for another 1 h. The polymerized GMPCPP-microtubules were pelleted by 90,000 rpm for 10 min at 37 °C (TLA120.1; Beckman Coulter) and suspended in warm (37 °C) EM buffer.

### Cryo-EM grid preparation and imaging

GMPCPP-microtubules were diluted to ∼0.25 mg/ml in EM buffer. Microtubules were applied to a glow discharged Quantifoil R1.3/1.3 300 mesh copper grid (Electron Microscopy Sciences, 350CR1.3) in the chamber of a Vitrobot (Thermo Fisher) set to 25 °C and 100% relative humidity. Microtubules were allowed to adhere to the grid for 30 s, and the grid was then blotted for 4 s and plunged into ethane. Tubulin dimers were prepared in 25 mM Tris (pH 7.5), 200 mM NaCl, 1 mM MgCl_2_, and 1 mM EGTA and were diluted to 2 mg/ml and applied to the cryo-EM grid in the same manner, however, without a wait time. Micrographs were collected on a Talos Arctica electron microscope operated at 200 kV equipped with Gatan K2 direct electron detectors. Micrographs were collected in the counting mode using Leginon (66) with a nominal magnification of 45,000×, giving a final pixel size of 0.91 Å per pixel. 40 frames of 200 ms each were collected with a defocus range from −1.24 to −1.72 μm at a dose rate of 5.373 e^−^/Å^2^/sec for a total dose of 42.90 e^-^/Å^2^. MotionCorr 2.0 (67) was used for frame alignment.

## Data availability

All data are contained in the article with the exception of the raw mass spectrometry data that are deposited at https://osf.io/m62x7/.

## Supporting information

This article contains supporting information.

## Conflict of interest

The authors declare that they have no conflicts of interest with the contents of this article.
