# Supplementary material for: Molecular determinants for α-tubulin methylation by SETD2
Source: J Biol Chem. 2021 Jun 19;297(1):100898. doi: 10.1016/j.jbc.2021.100898 (PMC8294582; doi:10.1016/j.jbc.2021.100898)
Supplement: Figures S1–S5 and Table S1 [file mmc1.docx]

**Supplemental Figures**


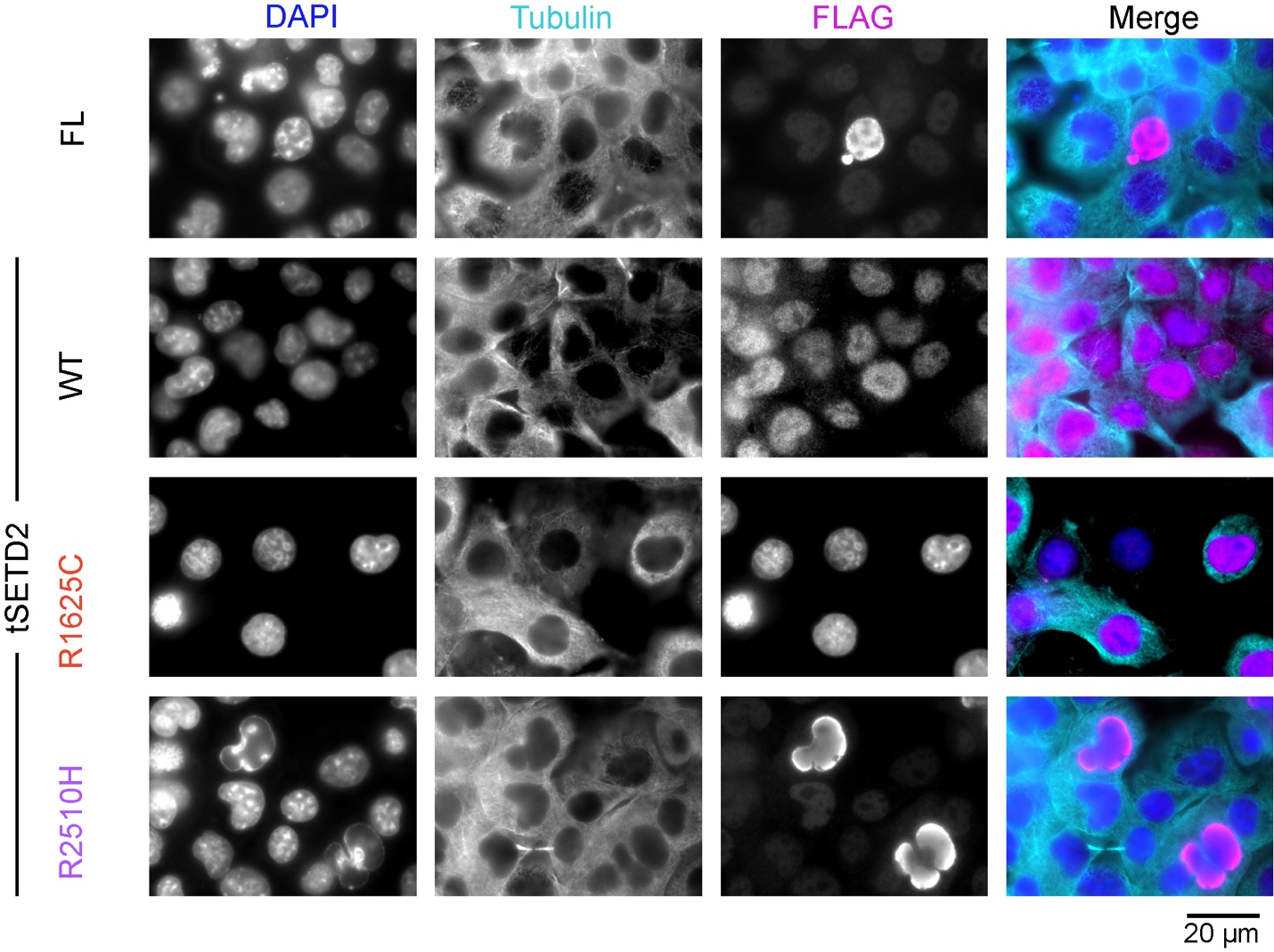


**Figure S1: Localization of tSETD2-FLAG constructs in interphase cells.** COS-7 cells transiently expressing (top row) FL SETD2-FLAG, (second row) tSETD2-FLAG, (third row) tSETD2(R1625C)-FLAG or (bottom row) tSETD2(R2510R)-FLAG were fixed and stained for DAPI (DNA, dark blue) and with antibodies against β-tubulin E7 (teal), and the FLAG tag (magenta).


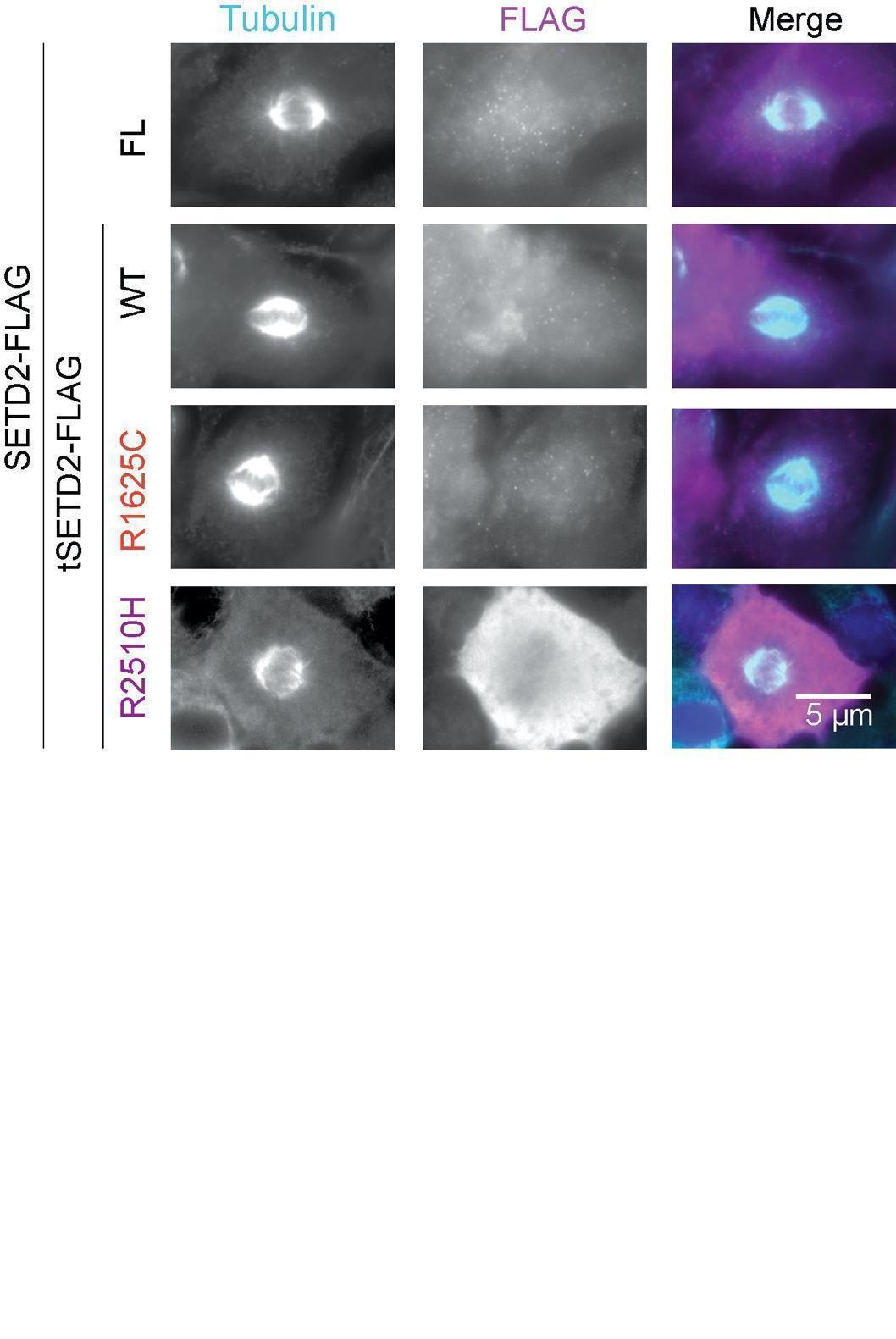


**Figure S2: Localization of tSETD2-FLAG constructs in mitotic cells.** COS-7 cells transiently expressing (top row) FL SETD2-FLAG, (second row) tSETD2-FLAG, (third row) tSETD2(R1625C)-FLAG or (bottom row) tSETD2(R2510R)-FLAG were fixed and stained with antibodies against α-tubulin (blue), and the FLAG tag (magenta).


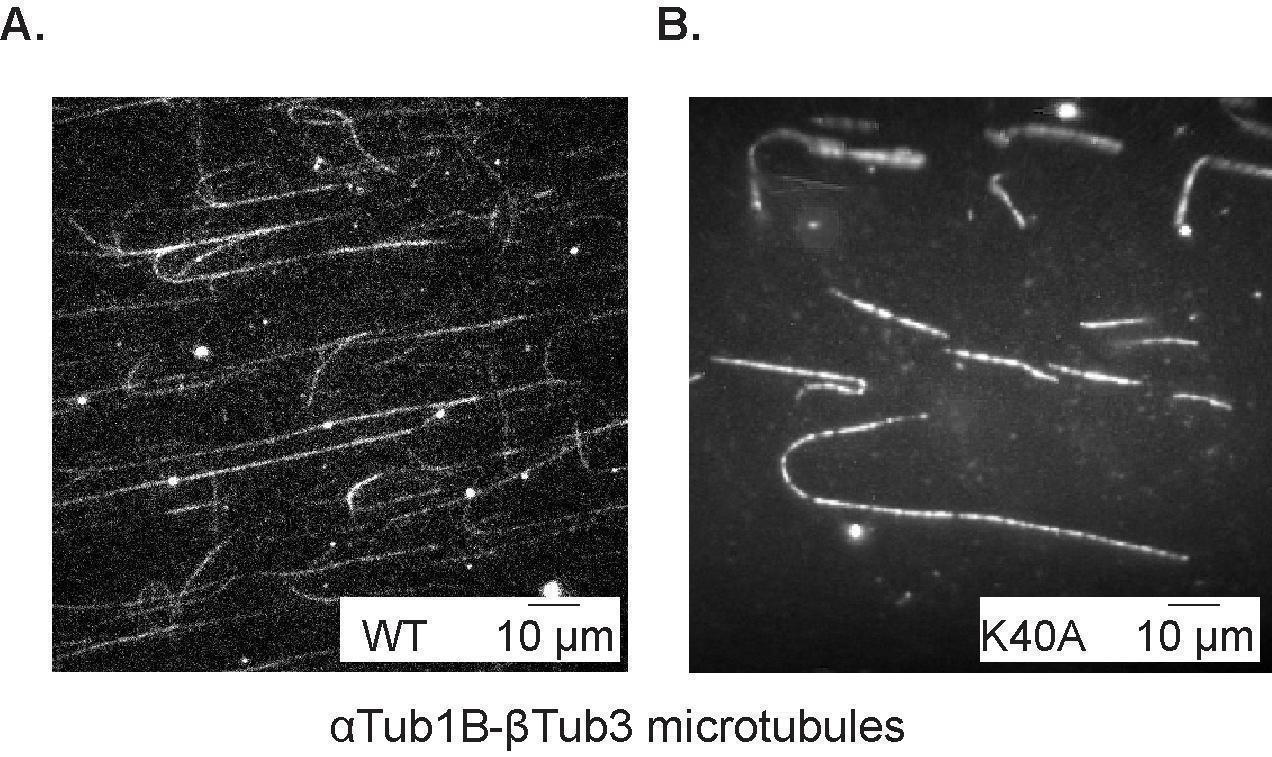


**Figure S3: Polymerization of recombinant single-isotype tubulin into microtubules.** Recombinant A) WT αβ-tubulin or B) mutant αβ-tubulin(αK40A) purified from insect cells was incubated with GMPCPP seeds to nucleate polymerization. To visualize growing microtubules, the reactions were spiked with 2% of 488- and biotin-labeled porcine brain tubulin. Shown is a representative field of view imaged by TIRF microscopy.


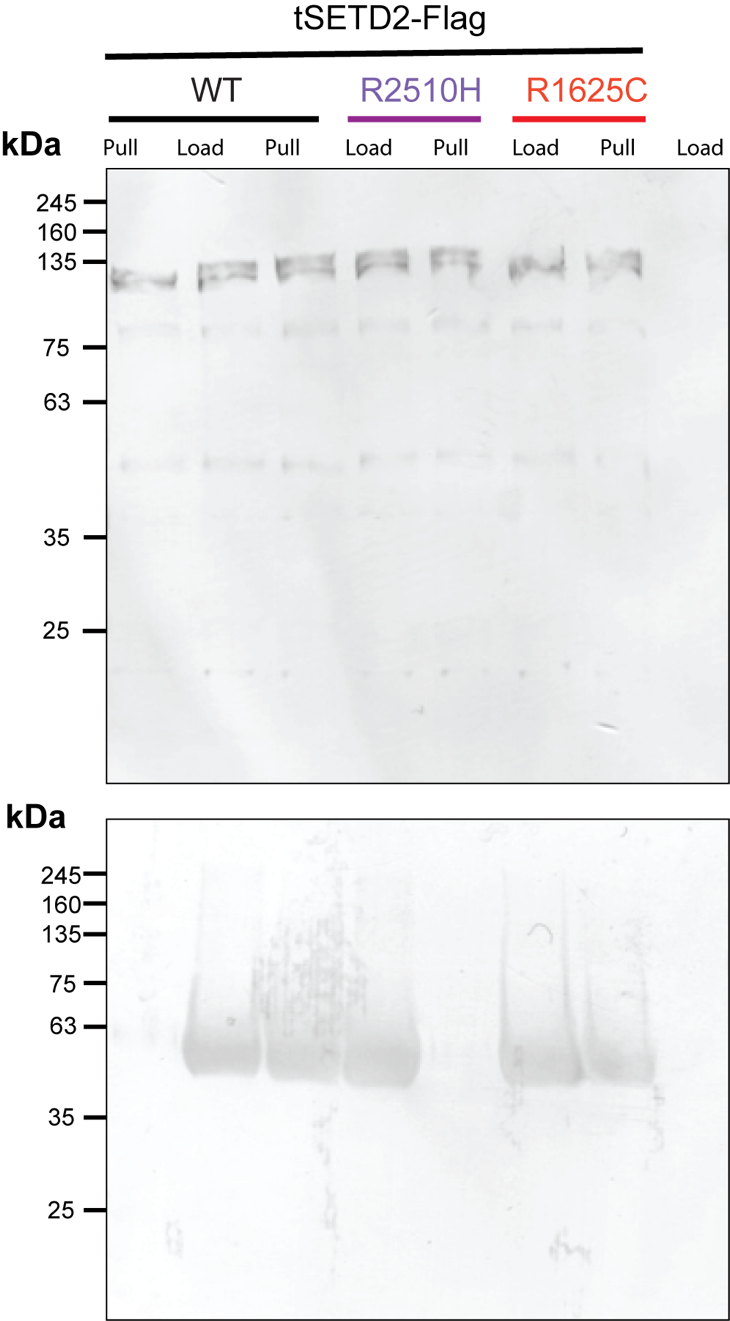


**Figure S4: Related to Figure 4D**. Uncropped view of western blots with top) anti-FLAG and bottom) anti-tubulin E7 antibodies.


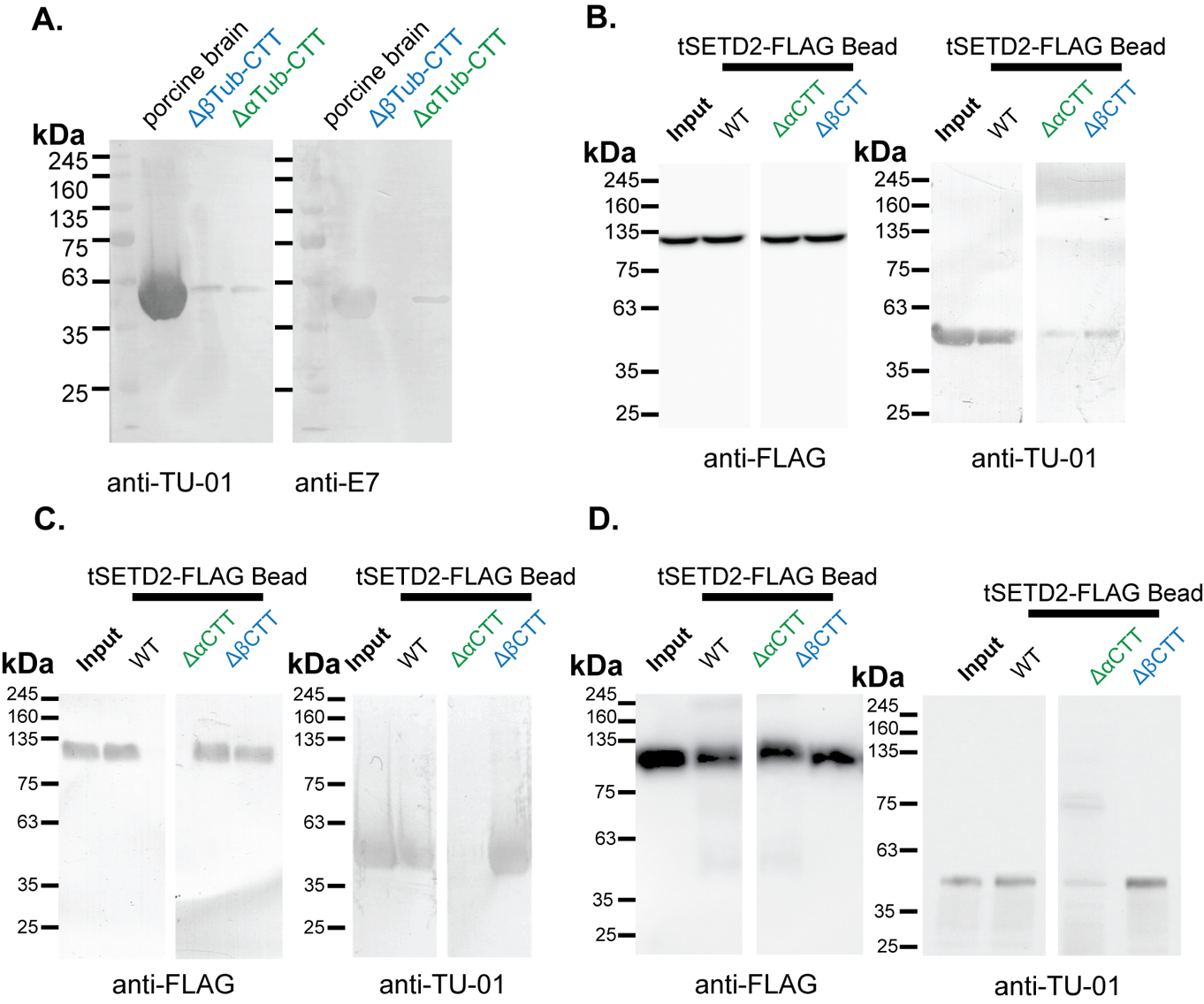


**Figure S5: Related to Fig. 6.** A) Uncropped view of western blots with antibodies to the left) α-tubulin N-terminus (TU-01) or right) β-tubulin CTT (E7). B-D) Uncropped view of IP western blots with antibodies to left) the FLAG tag and right) α-tubulin N-terminus (TU-01).

**Table S1:** Metadata of mass spectrometry analysis of the reaction of tSETD2-FLAG with single-isotype tubulin. Please find raw data: https://osf.io/m62x7/

| Peak-generating software | Proteome Discoverer, v2.3 (ThermoFisher) |
| --- | --- |
| Sequence database searched | UniProt human protein database (downloaded on 06/20/2019; 20353 reviewed entries) |
| Specificity of proteases | Chymotrypsin |
| Number of missed and/or non-specific cleavages permitted | 2 |
| List of all fixed modifications considered (including residue specificity) | Carbamidomethylation on Cys |
| List of all variable modifications (including residue specificity) | Deamination of Asn/Gln, oxidation of Met, methylation and acetylation of Lys |
| Mass tolerance for precursor ions | 10 ppm |
| Mass tolerance for fragment ions | 0.1 Da |
| Threshold score/expectation value for accepting individual spectra | Percolator, a semisupervised machine learning PSM validator node in Proteome Discoverer v2.3 software, was used to discriminate between correct and incorrect spectrum identification. |
| Estimation of false discovery rate and how calculated (for large datasets) | False discovery rate (FDR) was determined using Percolator and proteins/peptides with a FDR of ≤1% were retained for further analysis |
